# Pattern Recognition in Bioinformatics

**DOI:** 10.1155/2016/5284169

**Published:** 2016-10-09

**Authors:** Sher Afzal Khan, Daojing He, Jose C. Valverde

**Affiliations:** ^1^Faculty of Computing and Information Technology in Rabigh, King Abdul Aziz University, Jeddah, Saudi Arabia; ^2^Department of Computer Science, Abdul Wali Khan University Mardan, Pakistan; ^3^Department of Computer Sciences, South China University of Technology, Guangzhou, China; ^4^School of Computer Science and Software Engineering, East China Normal University, Shanghai, China; ^5^Department of Mathematics, University of Castilla-La Mancha, Albacete, Spain; ^6^Institute of Applied Mathematics in Sciences and Engineering, Ciudad Real, Spain

Every accumulation of data in its raw form holds obscure patterns. Pattern recognition deals with the science of transforming and classifying entities on the basis of these patterns. It is a vast field as it deals with data from diverse sources. Data can be of single dimensional nature as in case of stock exchanges and sound, two-dimensional as in case of images, and even multidimensional. It has many applications, for example, in medical science, it provides origins for computer-aided diagnosis (CAD) which supports medical practitioners in interpretations and finding of diseases. It has other typical applications: automatic speech recognition; recognition of text in various categories; and automatic recognition of human faces. Moreover, the genetic and protein structure in living organisms form intrinsic patterns. Data collected from the decomposition of these proteins help to identify them and hence to classify the protein. The ultimate objective is to make machines ideally as intelligent as humans in recognizing such patterns which help to form automated systems for conduction of routine matters.

Bioinformatics deals with development of algorithms and software for understanding the biological data. For analyzing and interpretation of the biological data, bioinformatics uses mathematics, statistics, computer, and engineering. There exists a lot of work in molecular biology using various approaches of bioinformatics like image processing and machine learning.

Bioinformatics not just deals with application of pattern recognition for protein classification but it also incorporates use of computational intelligence in protein sequencing, gene expression, comparative genomics, mutation, disease genetics, and molecular interactive networks.

In this special issue, we focused on innovation of cutting edge technology in the fields of pattern recognition and bioinformatics with multidimensional scope.

The articles published in this issue contain various aspects of PARE. Y. Ren et al. propose an algorithm Autoregressive Bayesian spectral Regression (ABSR) to estimate rhythmicity of a gene expression profile with short time series. G. Zhao et al. propose a supervised learning-based Chinese Visible Human (CVH) brain tissues segmentation method that uses stacked autoencoder (SAE) to automatically learn the deep feature representations. A. Butt et al. present a computationally intelligent technique used for the prediction of membrane protein. They use statistical moments for extracting features. Furthermore, for prediction of membrane protein, multilayer neural network is trained based on backpropagation. S. Qadri et al. describe pattern recognition for the classification of five land cover patterns data from remote sensing images. These land patterns are used quantitatively in the form of texture and multispectral data. Moreover, selection techniques and artificial neural network are used for selection of texture features and for classification land cover patterns data, respectively.

The work by Nguyen et al. performs a number of quantitative and structure-based analyses including hydrophobic percentage calculation, structural modeling, and molecular docking study of bacteriocins of interest against protein p53, a cancer target.

U. Jamil et al. use an automatic approach to preprocessing the image and then segment the skin lesion. The main aim is to find the accurate result to diagnose melanoma, the 5th form of skin cancer. The automatic system can filter unwanted artifacts including hairs, gel, bubbles, and specular reflection. The approach is further tested and compared and found the better result.

